# Facial Orientation and Facial Shape in Extant Great Apes: A Geometric Morphometric Analysis of Covariation

**DOI:** 10.1371/journal.pone.0057026

**Published:** 2013-02-18

**Authors:** Dimitri Neaux, Franck Guy, Emmanuel Gilissen, Walter Coudyzer, Patrick Vignaud, Stéphane Ducrocq

**Affiliations:** 1 Institut de Paléoprimatologie, Paléontologie Humaine : Evolution et Paléoenvironnements, Université de Poitiers, CNRS UMR 7262, Poitiers, France; 2 Department of African Zoology, Royal Museum of Central Africa, Tervuren, Belgium; 3 Université Libre de Bruxelles, Laboratory of Histology and Neuropathology, Brussels, Belgium; 4 University of Arkansas, Department of Anthropology, Fayetteville, Arkansas, United States of America; 5 University Hospitals Leuven, Department of Radiology, Leuven, Belgium; Team ‘Evo-Devo of Vertebrate Dentition’, France

## Abstract

The organization of the bony face is complex, its morphology being influenced in part by the rest of the cranium. Characterizing the facial morphological variation and craniofacial covariation patterns in extant hominids is fundamental to the understanding of their evolutionary history. Numerous studies on hominid facial shape have proposed hypotheses concerning the relationship between the anterior facial shape, facial block orientation and basicranial flexion. In this study we test these hypotheses in a sample of adult specimens belonging to three extant hominid genera (*Homo*, *Pan* and *Gorilla*). Intraspecific variation and covariation patterns are analyzed using geometric morphometric methods and multivariate statistics, such as partial least squared on three-dimensional landmarks coordinates. Our results indicate significant intraspecific covariation between facial shape, facial block orientation and basicranial flexion. Hominids share similar characteristics in the relationship between anterior facial shape and facial block orientation. Modern humans exhibit a specific pattern in the covariation between anterior facial shape and basicranial flexion. This peculiar feature underscores the role of modern humans' highly-flexed basicranium in the overall integration of the cranium. Furthermore, our results are consistent with the hypothesis of a relationship between the reduction of the value of the cranial base angle and a downward rotation of the facial block in modern humans, and to a lesser extent in chimpanzees.

## Introduction

Among the numerous skeletal modifications which have occurred during hominin evolution (the term ‘hominin’ refers to the members of the human clade and the term ‘hominid’ corresponds to the common ancestor of *Homo*, *Pan*, *Gorilla* and *Pongo* and all of its descendants), the morphology of the face has undergone several important changes (see e.g. [1] or [2] for studies on early fossil hominin). These changes are particularly notable for facial projection, i.e. the degree to which the face projects in front of the cranial base [3] and facial prognathism, i.e. the protrusion of the lower face relative to the upper face [4]. These modifications of facial characteristics raise several important questions. They concern notably the role that the integration between the face and other parts of the cranium, such as the basicranium and the neurocranium, plays in facial morphological changes [5]–[7]. Hence, the study of the relationships between these structures is crucial for a better understanding of the set up of facial morphology, e.g. the reduction of facial prognathism in the *Homo* genus. Thus, several studies have already focused on the relationship between the face and other parts of the cranium [3], [8]. They bear, for example, on the link between facial orientation and basicranial flexion [9], [10], or on the relationship between facial projection and sphenoid length [11]. The development of geometric morphometric (GM) methods has significantly contributed to the study of facial shape morphology in hominins and hominids. Several studies have employed this technique to better understand the relationship between the face and other cranial characteristics. Two-dimensional GM analyses on samples of modern humans produced new data with respect to covariation of facial shape with the neurocranium [12] and with the lateral basicranium [13], [14]. Using three-dimensional GM, Mitteroecker and Bookstein [15] were able to demonstrate that *Pan*, *Gorilla* and *Homo* possess similar patterns of evolutionary integration between the face and neurocranium, although certain characteristics in modern humans evolved in a less-integrated way. Hallgrímsson and Lieberman and colleagues [16], [17] and Hallgrímsson and colleagues [18] have used a combination of GM and linear measurements on mouse crania as a proxy to understand the developmental pathways that express the relationships between primate facial shape and basicranium flexion.

Despite all these studies that have bearing on integration of the facial shape, several aspects remain unclear including the relationship between: on one hand (1) the facial shape, i.e. the morphology and proportion of the anterior face, and on the other hand (2) the cranial base flexion and (3) the orientation of the so-called “facial block” hypothesized by Lieberman and colleagues [3] and McCarthy and Lieberman [8], i.e. the orientation of the group of structures which comprises the frontal lobes, the anterior cranial fossa (ACF) and floor and the ethmomaxillary complex, relative to the basicranium. However, the importance of this character has previously been suggested by other authors [3], [19]. Lieberman and colleagues [3], [4] defined two major constraints that play a part in the relationship between the basicranial flexion and the orientation, rotation, and projection of the facial block: (1) the roof of the orbits is also the floor of the ACF (orbital part of the frontal bone and lesser wings of the sphenoid), and (2) the junction between the middle cranial fossa (MCF) and the maxilla (ethmomaxillary complex) is almost always perpendicular to the neutral horizontal axis (NHA) of the orbits [8], [20]. The result of these two constraints is that the whole face should rotate with the ACF as a block, or a unit. Thus, an extension of the ACF relative to the posterior cranial fossa (PCF), i.e. an increase of the value of the cranial base angle (CBA), should lead to a mechanical upward rotation of the facial block. Conversely, a flexion of the ACF relative to the PCF, i.e. a decrease of the value of the CBA, should lead to a rotation of the facial block beneath the ACF (downward). The arguments of the authors were based on interspecific comparisons in anthropoids [3]. In our study, we assess, at the intraspecific level, the relationship proposed by Lieberman and colleagues at the interspecific level [3] by exploring the variation of the facial block orientation and basicranial flexion within species, in a sample of adult extant hominids (*Homo*, *Pan* and *Gorilla*).

Lieberman and colleagues [3] also claim that while it is clear that flexion of the basicranium plays a major role in influencing the facial block orientation, there is less information about the potential influence of the cranial base on other aspects of facial shape (i.e. height, width, shape and organization of structures within the face). Enlow and Hans [19] have proposed that a long, narrow (dolichocephalic) and weakly-flexed basicranium should be correlated to an anteroposteriorly and vertically-elongated, i.e. supero-inferiorly longer, anterior face. In our study, we focus on the relationship between anterior facial shape, facial block orientation and basicranial flexion and length by evaluating the strength and the patterns of the correlation between these features [21]. In this paper, we first appraise (1) whether or not Lieberman's hypothesis [3] of a reduction of the CBA linked to downward rotation of the facial block holds in modern humans and in *Pan* and *Gorilla* by assessing the variation of these morphological structures, and (2) then we test if there is a significant relationship between the anterior facial shape and facial block orientation, linked to cranial base flexion and, if this is the case, whether it follows Enlow and Hans' [19] hypothesis of a vertically-elongated face linked to a long and weakly-flexed basicranium.

## Materials and Methods

### Definitions

The face is constituted of several interrelated bones that surround a diverse set of organs and spaces, e.g. the orbits, the pharynx, the nasal and oral cavity [4]. Anatomically, the mandible belongs to the face, but the relationships between the mandible and the cranium are complex and require that the relationship between the basicranium and the mandibular ramus be taken into account [13], [14], [22], [23]. However, this is beyond the present scope of this study, which focuses on the cranial part of the face. In this study, the term “upper face” corresponds to the part of the face above the rhinion; the “middle face” is between the rhinion and the anterior nasal spine; and finally, the “lower face” is the area below the anterior nasal spine.

**Figure 1:**
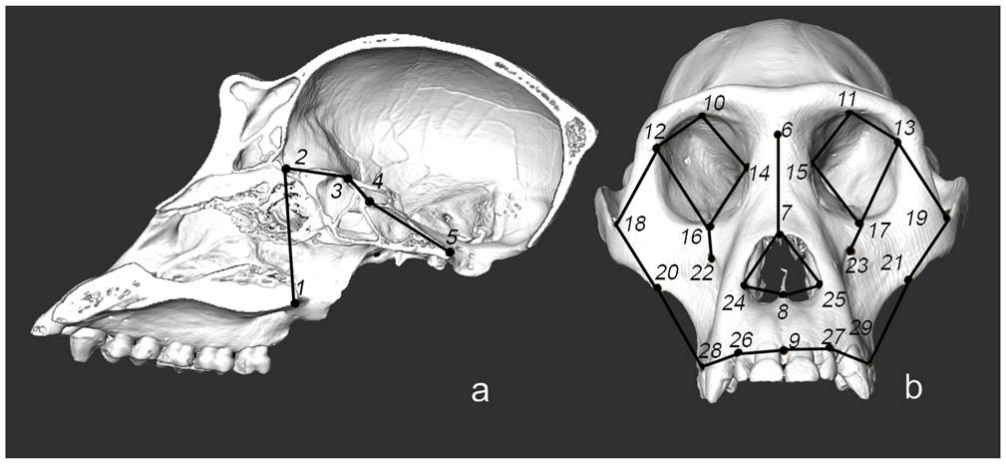
Landmark locations on the cranium of a female *Gorilla* **.** a. Set 1. Sagittal cut showing facial and basicranial flexion points (1–5). b. Set 2. Frontal view showing facial shape points (6–29).

According to Lieberman and colleagues [3] and McCarthy and Lieberman [8], the facial block is composed of the frontal lobes, the anterior cranial base and floor and the ethmomaxillary complex, which includes the ethmoid, maxilla and palatine. These authors used the posterior maxillary (PM) plane, defined as the midsagittal projection of a line from the maxillary tuberosities to the anterior poles of the MCF, in order to describe facial orientation [8], [20]. Since the PM plane is based on the midsagittal projection of two lateral landmarks [8], [20] and since the patterns of integration of midsagittal and lateral face may differ as it is the case in the basicranium [13], [24], we choose to defined the orientation of the facial block using midsagittal landmarks, i.e. the staphylion, the foramen caecum and the sphenoidale (Fig. 1.a, Table 1), instead of the PM plane. Staphylion is the midsagittal infero-posterior limit of the facial block and the foramen caecum is the anterior most point of the ACF, that is in direct anatomical contact with the upper face [3] and which is a growth counterpart of the face [4], [19]. These two points permit the measurement of the facial block orientation without needing to take the modifications due to anterior face morphology into account, since this anterior face is prone to variation such as prognathism or to the development of supraorbital torus. Thus, in this study, we focus on the midsagittal orientation of the facial block using midsagittal anatomical landmarks. In order to appraise for the differences between these landmarks, used in our analysis, and the PM plane, we measure how much the staphylion-sphenoidale chord (StSp) deviates from the close to 90° relationship to the neutral horizontal axis (NHA) of the orbits that the PM plane follows [8], [20], [25]. We also test whether the angles PM-NHA and StSp-NHA and the angle between the staphylion-foramen caecum chord and the NHA (StFc-NHA) are statistically different across *Homo*, *Pan* and *Gorilla*.

**Table 1:** Landmark definitions.

Count	Landmark	Definition
1	Staphylion	The point on interpalatal suture corresponding to deepest point of notches at the rear of the palate.
2	Foramen caecum	Most anterior inferior point of anterior cranial base
3	Sphenoidale	Most superior and posterior midline point on the tuberculum sellae
4	Sella	Point (in space) at the center of the sella turcica
5	Basion	Most anterior and inferior midline point on the margin of the foramen magnum
6	Nasion	Midline intersection of nasal and frontal bones
7	Rhinion	Midline point at the inferior end of the internasal suture
8	Nasospinale	Most anterior point on nasal spine.
9	Prosthion	Most anterior midline point of the maxillary alveolar process
10, 11	Superior margin of orbit	Midline point of the superior margin of the orbit.
12, 13	Frontomalare orbitale	Point where the frontozygomatic suture crosses the inner orbital rim.
14, 15	Dacryon	The most superior point at which the lacrimomaxillary suture
meets the frontal bone.		
16, 17	Zygoorbitale	Point at which the zygomaticomaxillary suture meets the orbital rim.
18, 19	Jugale	Point in the depth of notch between the temporal and frontal
processes of the zygomatic bone.		
20, 21	Zygomaxillare	The most inferior point of the zygomaticomaxillary suture.
22, 23	Infraorbital foramen	Measured at the centre, in the plane of the bone surface.
24, 25	Alare	The most lateral point on the margin of the nasal aperture.
26, 27	Alveolar I2	Point in the centre of the extern border of the I2 alveolus
28, 29	Alveolar P3	Point in the centre of the extern border of the P3 alveolus

1–5: facial positioning. 6–29: facial shape.

Definitions adapted from [31], [33], [39].

The basicranial flexion, often quantified by the CBA, characterizes the flexion of the ethmoid bone, sphenoid bone and basilar part of the occipital bone and therefore, the relative position of anterior, middle and posterior cranial fossa [26]–[28]. In this study it is defined by the foramen caecum, the sphenoidale, the sella and the basion. As described, this representation of the basicranial flexion is closed to the CBA1 [28], [29]. This definition, which includes the foramen caecum, is recommended for the study of the relationship between the basicranium and the face, as the foramen caecum is included in the ACF which is in direct contact with superior part of the face by the orbital part of the frontal bone and the little wing of the sphenoid bone [27].

In this study, in the midsagittal plane, the ACF is considered from the foramen caecum to the sphenoidale, the MCF from the sphenoidale to the sella and the PCF from the sella to the basion. It follows Lieberman's [4] definition except for the dorsum sellae, replaced by the sella turcica (Fig. 1.a, Table 1).

### Material

The study used a sample of 129 extant hominid crania, including 68 modern humans (*Homo sapiens*) including 34 females and 34 males. The chimpanzee sample consisted of 33 common chimpanzees (*Pan troglodytes*) including 17 females and 16 males. Finally 28 gorillas (*Gorilla gorilla*) including 14 females and 14 males were studied. All specimens were considered as adults (i.e. third molars erupted).

#### Specimen Acquisition

Crania are housed in various European institutions: the Royal Museum for Central Africa (Tervuren, Belgium), the Anthropologisches Institut und Museum (Zürich, Switzerland) and the Natural History Museum (London, United Kingdom). Depending on their location, specimens were scanned either in the Department of Radiology in Universitair Ziekenhuis (UZ) in Leuven (Belgium), in the Kantonsspital in Winterthur (Switzerland), or in the Hammersmith Hospital in London (United Kingdom). Each cranium was scanned using a medical computerized tomography (CT) scanner, with a pixel size and a slice thickness adjusted according to specimen cranial size. Pixel size ranged from 0.30 mm (chimpanzees) to 0.70 mm (gorillas, humans) and slice thickness from 0.30 mm (chimpanzees) to 1.0 mm (humans). CT images of each specimen were acquired by two of us (F.G. and W.C.). CT-scan data were computed using Avizo v6.0 software (©Visualization Sciences Group). Bone material was extracted from the virtual volume using automatic thresholding. For dry specimens, this step is relatively straightforward since they present either material information (bone) or empty space. A bone protocol, emphasizing hard versus soft tissues was applied during the scan session for the wet specimens allowing only minor manual corrections to the automatic segmentation for a complete extraction of the bone information. The corrected volumes were then converted into 3D surfaces for the purposes of our analysis.

#### Ethics statement

Human material consists in anonymized CT-scan images of non identifiable bone tissues.

### Data acquisition

#### Landmarks

We characterized the morphology of the anterior face, facial block position and basicranial flexion using 3D landmark coordinates. Two set of landmarks were defined: Set 1 includes 5 facial and basicranial landmarks illustrating the orientation of the facial block and the flexion of the cranial base as traditionally defined in the literature [29]. Facial block orientation and basicranial flexion are defined in the midsagittal plane.

Set 2 includes 24 midsagittal (4) and bilateral (20) landmarks characterizing the morphology of the anterior face (Fig. 1.b, Table 1). They are situated in a three-dimensional space in order to best represent the facial skeletal shape. The chosen landmarks give precise data on facial elongation, facial width and proportion and on facial structure organization such as orbit or nasal aperture locations, as in other studies [30]–[33].

Landmarks were placed on three-dimensional surfaces with the Landmark v3.0 software [34]. In order to avoid possible complications stemming from bilateral asymmetry, we chose to compute a symmetrical configuration from original landmarks coordinates [35], [36]. The use of the symmetric shape component is useful to reduce dimensionality in datasets where variables exceed sample size [37]. All the virtual crania are identically oriented, the original configuration was hence duplicated and the resulting configuration reflected (i.e., all the left landmarks were transformed to become right landmarks and vice versa) using the R software [38]. The reflected and the original configurations were averaged. Therefore, we obtained a perfectly symmetrical configuration that discounts fluctuations due to bilateral asymmetry. Computation from original to symmetrical configuration shows minimal (non- significant) point deviation.

### Data analysis

#### Raw data

Analyses were carried out using three-dimensional GM data that facilitate detailed assessment of the anterior facial shape variations and relationships to facial block orientation [31], [39]. The symmetrical configuration of landmarks coordinates was subjected to GM in order to depict relationships between facial shape and position [40], [41]. This widely-used technique allows the quantification and description of the morphological variations within a set of specimens [15], [22], [29]–[32], [35], [42]–[44]. Additionally, GM analyses allow size and shape to be assessed independently [45].

#### Angles comparison

In order to appraise for the differences between the midsagittal landmarks used in our analysis and the PM plane, the difference between the values of PM-NHA and StSp-NHA was measured. NHA is defined as the segment between (1) the midsagittal projection of the supero-inferior midpoint between the lower and upper orbital rims and (2) the supero-inferior midpoint between the superior orbital fissures and the inferior rims of the optic canals [8], [20]. We performed an ANOVA to test for significant differences between PM-NHA and StSp-NHA values in each taxon. Using ANOVA, we also test if the values of each angle (PM-NHA, StSp-NHA and StFc-NHA) are significantly different across *Homo*, *Pan* and *Gorilla*.

#### Overall interspecific variation

Morphologika v2.5 software was used to perform a Procrustes superimposition [40], [41] and a principal component analysis (PCA) [45], [46] including all the taxa and using landmarks of set 1 and set 2, pooled together. This PCA allowed assessing the overall interspecific variation in the sample and the distribution of individuals in the shape space [47].

#### Facial orientation

A second Procrustes superimpositions and PCA was performed within each taxon. Here, the Procrustes superimposition was performed for each set of landmarks within each taxon. Procrustes coordinates of set 1 and set 2 were computed independently. PCA was used to assess the intraspecific variations of facial block orientation and basicranial flexion in order to assess Lieberman's hypothesis.

#### Allometry and variance dependence of integration

Allometry is a factor that might influence patterns of morphological integration [48]. Previous studies highlighted the covariation of facial size with the rest of the cranium, particularly the cranial base [29], [49]. Furthermore, static allometry [50] can be expected between male and female specimens [51], [52]. For this reason, we tested the influence of size on each set of landmarks for each taxon in our study. We used multivariate regressions of Procrustes coordinates on the logarithm of centroid size (log CS) [53] for each taxon, using MorphoJ v1.02 software to test for potential influence by allometry [54]. Centroid size is defined as the square root of the sum of squared distances of a set of landmarks from their centroid [41]. Multivariate regressions were performed independently for the first set of landmarks (facial block orientation), for the second set (anterior facial shape) and a third set of pooled landmarks. Within each taxon, a MANOVA is performed on the significant PC scores of the PCA of the regression residuals in order to test for differences in shape between sexes.

Integration may be dependant of variance, as an increase in the level of variance can result in an augmentation of the integration level [55], [56], [57]. In order to appraise for the integration linked to variance, we corrected our data for variance dependence of integration following Hallgrímsson et al. [55]. Corrected results are not significantly different from uncorrected previous analyses. Thus, for the sake of brevity, only results for uncorrected data are presented in this paper.

#### Craniofacial integration

Intraspecific covariance between facial block orientation and anterior facial shape was assessed by performing Partial least squares (PLS) analyses for each taxon. This method has been shown to be suitable for the study of covariation between two sets of variables (blocks) [12], [13], [15], [21], [29], [41], [45], [58], [59]. In our study two blocks were defined, which correspond to the two sets of Procrustes coordinates: block 1 (set 1) represents facial block orientation and block 2 (set 2) represents anterior facial shape (Fig. 1). The aim of the PLS is to maximize the covariance patterns between two blocks of variables rather than the intra-block variance. PLS describes data in terms of a score for each specimen along a single axis, similar to a principal component that is generated in a PCA. The primary difference is that, unlike principal components, which produces principal axes, PLS produces pairs of axes.

The PLS was performed between the two blocks using MorphoJ v1.02 software [54]. Since allometry can inflate measures of integration, the PLS analyses were recomputed using the residuals of the multivariate regression of shape variables on the logarithm of centroid size as variables. It allows the effect of size to be removed from the analyses [48]. We used the RV coefficient to measure the correlation resulting from the PLS [60]. The calculation of this coefficient is equivalent to the calculation of the correlation coefficient of a regression between two variables. The RV coefficient is a measure of the global integration between blocks. It ranges from zero to one with a zero value indicating that the two blocks are independent, and a value of one indicating that they diverge from one another only by a combination of rotation, translation and/or scaling [48], [61]. Although this approach does not strictly accept or reject a given hypothesis, it provides a global quantification of the strength of the association between blocks. Such an approach is recommended when studying covariation and indeed, there is a continuum between the complete absence of relationship and their complete covariation. Thus, the presence or absence of covariation does not represent discrete parameters [58], [62]. The use of the RV coefficient for the measure of the association between two blocks of variables has been recommended in several recent papers, notably because it is calculated directly on covariance and variance rather than on correlation values [48], [63]–[65].

## Results

### Angles comparison

The difference between the value of the angles PM-NHA and StSp-NHA is significant for *Homo* (8.9°±4.0; F_[1, 134]_ = 133.83, p<0.001) and *Pan* (5.9°±3.0; F_[1,64]_ = 36.16, p<0.001). It is at the limit of statistical significance for *Gorilla* (3.7°±3.1; F_[1,54]_ = 4.03, p = 0.05). The values of PM-NHA are not significantly different across the three species (F_[2, 126]_ = 1.25, p = 0.29) as well as the values of StFc-NHA (F_[2, 126]_ = 1.93, p = 0.15). At the contrary, the values of the angle StSp-NHA are significantly different across the three species (F_[2, 126]_ = 46.8, p<0.001).

### Principal components analysis (PCA)

#### Overall interspecific variation

**Figure 2:**
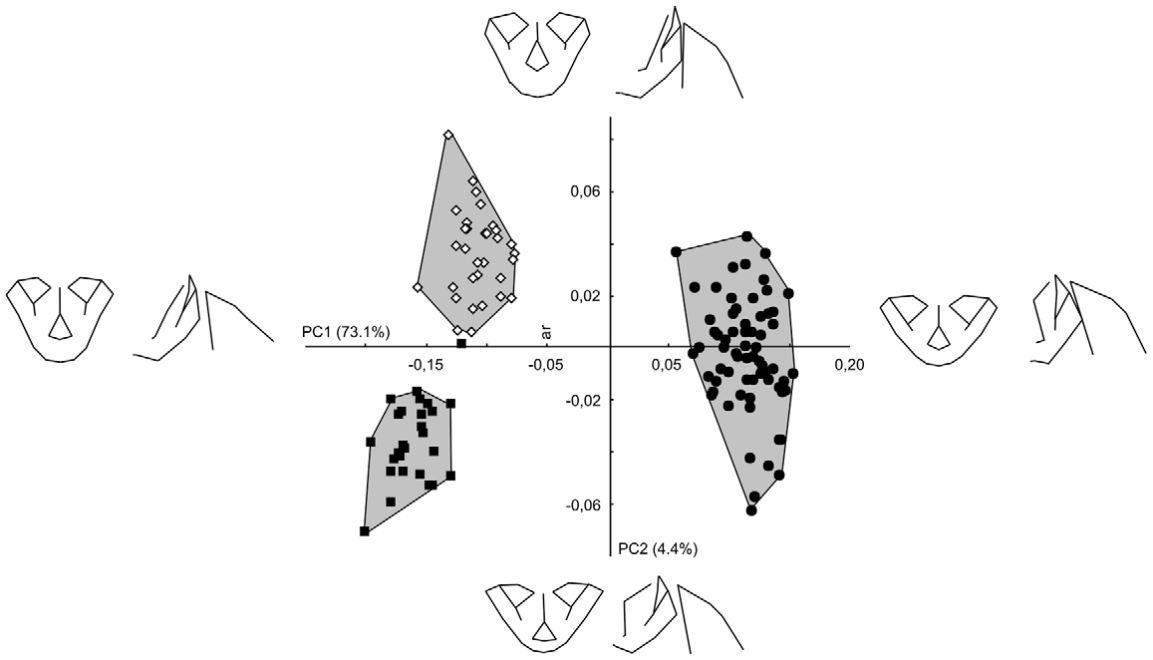
PCA including all the specimens and the landmarks of set 1 and set 2 pooled together. Wireframes represent, in frontal and sagittal view, the shape changes associated to an increase of 0.1 units of Procrustes distance. Full circles: moderns humans, empty diamond: chimpanzees, full squares: gorillas. Convex hulls gather specimens from each species.

**Figure 3:**
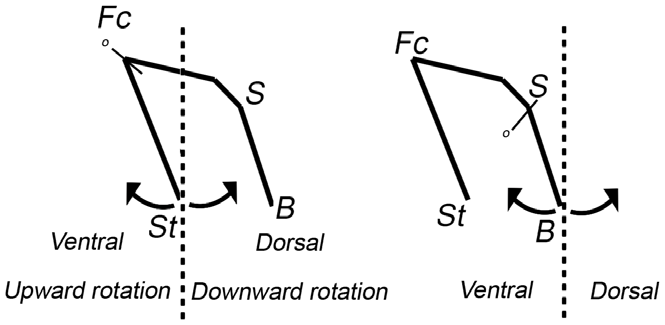
Example of the rotation of the staphylion (left) and of the basion (right). In this paper, rotation is considered as ventral if the distal end of the segment is displaced forward relative to the other end of the segment. It is considered as dorsal if it is displaced backward. St: staphylion, Fc: Foramen caecum, S: Sella turcica, B: Basion.

In the PCA performed on all the landmarks, including *Homo*, *Pan* and *Gorilla*, the first principal component (PC1) and the second principal component (PC2) explain 73.1% and 4.4% respectively of the total variance (Fig. 2). The first axis separates *Pan* and *Gorilla* on one hand and *Homo* on the other hand. Towards the higher scores on PC1, changes correspond to a shorter and wider face which is less prognathic and less projected relative to the ACF. The cranial base is more flexed and the facial block rotates dorsally (see Fig. 3). PC2 discriminates *Pan* and *Gorilla*. Towards the higher scores, the face is narrower and the lower face is superoinferiorly longer relative to the middle face. The middle face is also less projected anteriorly and the whole face is also less projected relative to the ACF. On this axis, there is a slight ventral rotation of the facial block but no significant modifications in the flexion of the cranial base.

#### Facial Orientation

**Figure 4:**
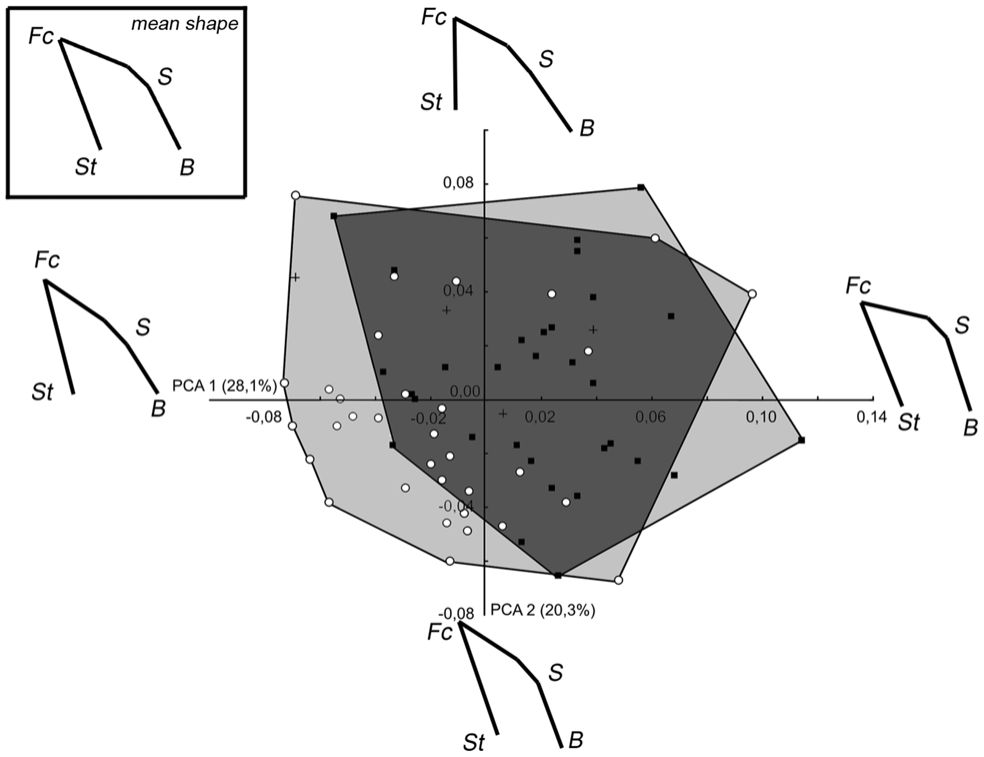
PCA_Hom: Set1 (facial block orientation) in modern humans. Wireframes represent, in sagittal view, the shape changes associated to an increase of 0.1 units of Procrustes distance. Empty circles: Female, full squares: Male. Convex hulls gather specimens from each sex. St: staphylion, Fc: Foramen caecum, S: Sella turcica, B: Basion.

When PCA is performed on the first set of landmarks in modern humans (PCA_Hom), PC1 and PC2 explain 28.1% and 20.3% respectively of the total variance (Fig. 4). Specimen distribution along the first two PC shows statistically significant distinctions between males and females (Wilk's λ = 0.759, F_[2,65]_ = 4.72, p<0.001). It can be observed that the majority of the female specimens fall to the lower left part of the graph. Towards the higher scores on PC1, changes correspond to a dorsal rotation of the facial block and to a ventral rotation of the anterior and posterior cranial base. These changes express a reduction of the CBA value associated with a dorsal rotation of the facial block. The main changes toward positive values along PC2 are a ventral rotation and a reduction of the height of the facial block caused by a relative forward displacement of the staphylion and a downward displacement of the foramen caecum. On this axis, a ventral rotation of the anterior cranial base and a backward displacement of the basion lead to an augmentation of the CBA.

**Figure 5:**
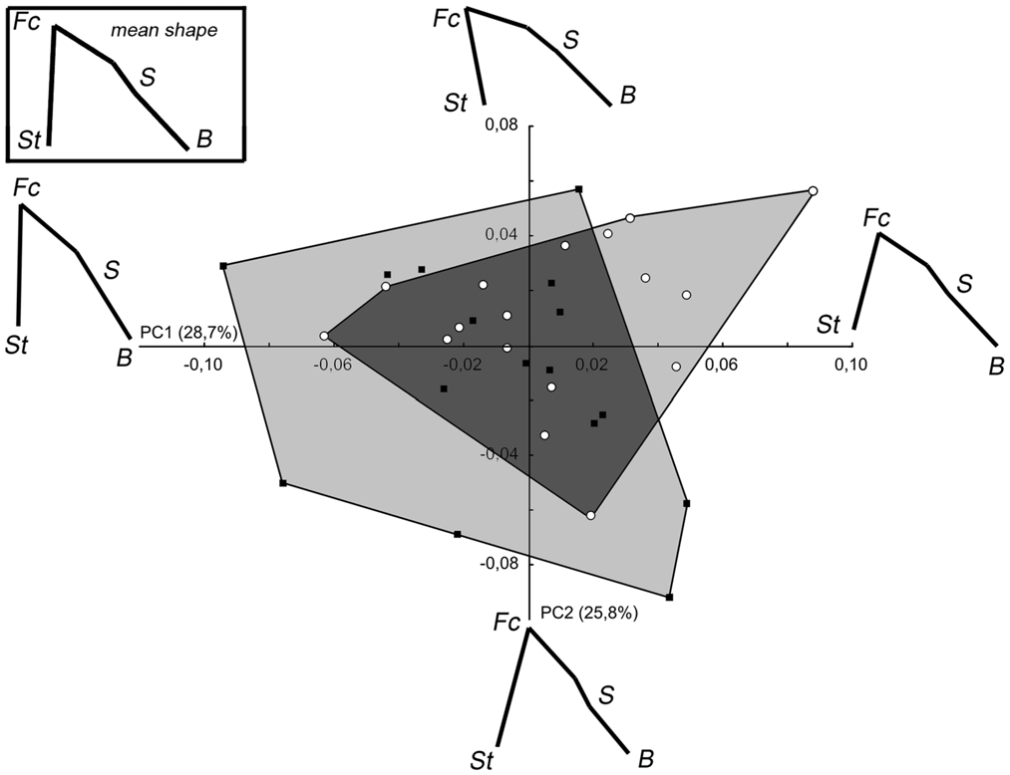
PCA_Pan: Set1 (facial block orientation) in *Pan*. For legend see figure 4.

In the PCA based on *Pan* specimens (PCA_Pan), PC1 and PC2 explain 28.7% and 25.8% respectively of the total variance (Fig. 5). On the first PC, males have lower score values and females tend towards the higher values, although both sexes overlap in a large part of this axis. For that reason, the distinction between males and females on the PC1-2 shape space is not significant (Wilk's λ = 0.870, F_[2,30]_ = 2.24, p = 0.124). This may also be due to the relatively small *Pan* sample size compared to the number of *Homo* specimens. On the first PC, the changes toward positive values represent a ventral rotation and a supero-inferior reduction of the facial block due to a forward displacement of the staphylion and a downward displacement of the foramen caecum. They are accompanied by a backward displacement of the basion leading to an increase in the value of CBA. Higher values on the PC2 indicate a dorsal rotation of the facial block, a ventral rotation of the anterior cranial base and a lower displacement of the basion resulting in a reduction of the CBA value.

**Figure 6:**
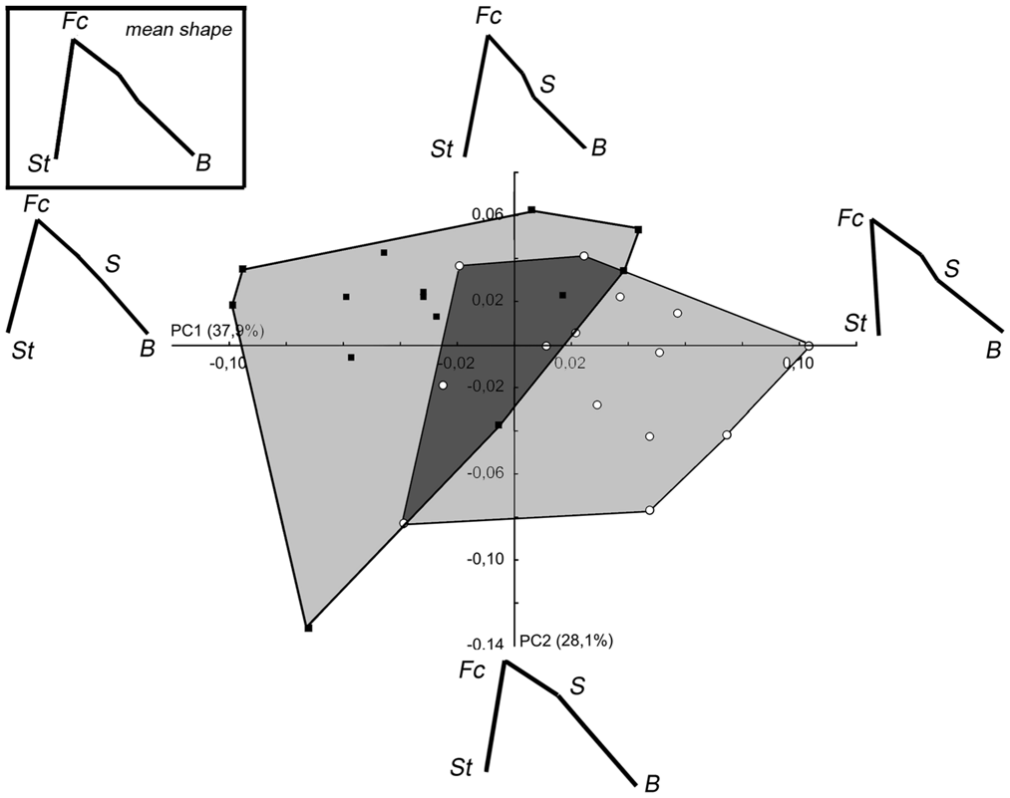
PCA_Gor: Set 1 (facial block orientation) in *Gorilla*. Convex hulls gather specimens from each sex. For legend see figure 4.

For the *Gorilla* analysis (PCA_Gor), PC1 and PC2 explain 37.9% and 28.1% respectively of the total variance (Fig. 6). As is observed for the *Pan* analysis (PCA_Pan), males have the lower score values and females the higher ones on the PCA1. However, the area where they overlap is reduced relative to the *Pan* analysis and the difference between both sexes is significant (Wilk's λ = 0.564, F_[2,25]_ = 9.64, p<0.001). In male gorillas, the facial block is more ventrally-rotated, the sella is more posteriorly positioned and the basion is superiorly positioned. On PC1, the higher scores indicate that there is a dorsal rotation of the facial block, a forward displacement of the sella and a downward displacement of the basion. On PC2, increasing positive values are associated with a ventral rotation and a reduction of the height of the facial block, which is linked to a marked forward displacement of the sella and a less marked forward displacement of the basion.

### Allometry

#### Allometry

The multivariate regressions of Procrustes coordinates (dependant variables) on size (Log CS – independent variables) show a significant influence of allometry for each taxon and for each set of landmarks (Table 2). For all the landmarks pooled together and for the first set of landmarks (facial orientation), allometry explains the least variance for *Homo* (respectively 5.6%, p<0.01; 6.8%, p<0.01) and the greatest variance for *Gorilla* (9.3%, p<0.01; 10.4%, p<0.01) and for *Pan* (11.1%, p<0.01; 11.9%, p<0.01). The second set of landmarks (facial shape) for *Gorilla* shows the most variance explained by allometry (18.9%, p<0.01) followed by *Pan* (10.7%, p<0.01) and *Homo* (3.7%, p = 0.02). MANOVA on PC scores of the PCA on the residuals reveals statistically different shapes between sexes in *Homo* for set 1 (Wilk's λ = 0.90, F_[2,65]_ = 3.77, p<0.05) and set 2 (Wilk's λ = 0.88, F_[3,64]_ = 2.92, p<0.05). Shape differences are not significant in *Pan* for set 1 (Wilk's λ = 0.92, F_[2,30]_ = 1.32, p>0.05) and set 2 (Wilk's λ = 0.79, F_[3,29]_ = 2.53, p>0.05) and in *Gorilla* for set 1 (Wilk's λ = 0.99, F_[2,25]_ = 0.94, p>0.05) and set 2 (Wilk's λ = 0.98, F_[3,24]_ = 0.17, p>0.05).

**Table 2:** Multivariate regressions of shape on size (lnCS) for all landmarks, set 1 (facial orientation) and set 2 (facial shape).

	Homo		Pan		Gorilla	
	Variance explained (%)	p-value	Variance explained (%)	p-value	Variance explained (%)	p-value
All	5.6	0.00	11.1	0.00	9.3	0.00
Set 1	6.8	0.00	11.9	0.00	10.4	0.00
Set 2	3.7	0.02	10.7	0.00	18.9	0.00

### Partial least squares (PLS)

#### *Homo*

**Figure 7:**
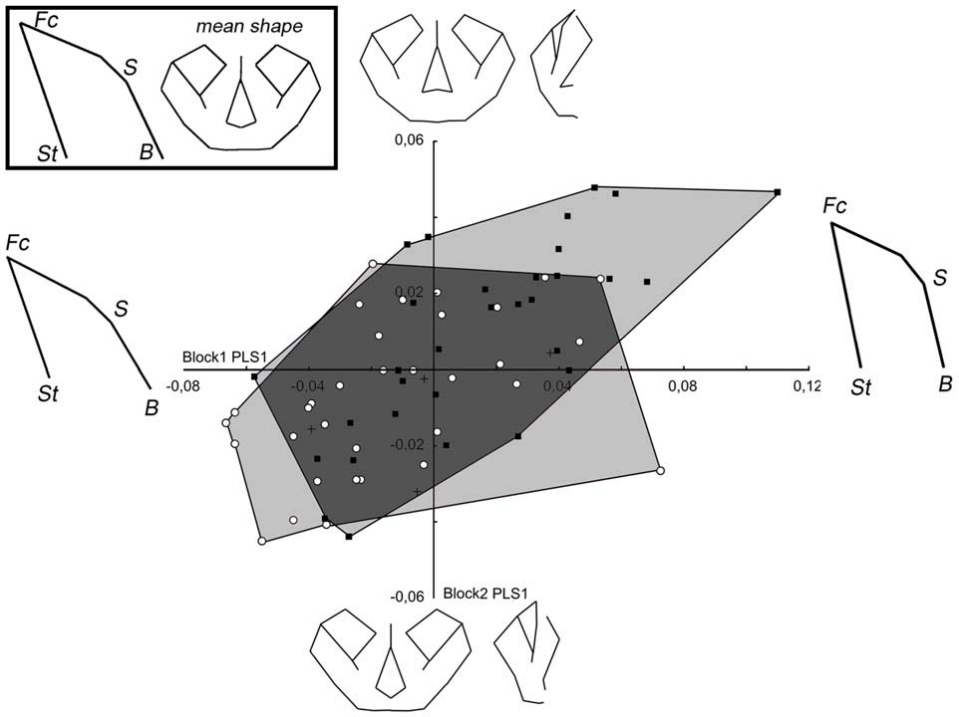
PLS of block 1 (facial block orientation) and 2 (facial shape) in modern humans. Wireframes show the shape changes along each singular axis. For legend see figure 4.

In the PLS analysis of modern humans, the first pair of singular axes accounts for 36.3% of the covariance (Fig. 7). The position of a specimen on the *x*-axis defines its shape relative to the first block (facial block orientation), while the position on the *y*-axis reflects the second block (facial shape). The RV coefficient indicates a significant relationship between the two blocks (RV = 0.15; p<0.01). Increasing positive values indicate a ventral rotation and an augmentation of the height of facial block, while a ventral rotation of the posterior cranial base are associated with a downward displacement of the lower face, an upward displacement of the central part of the upper face (nasion, rhinion, dacryon), and of the nasal spine, and an augmentation of lower face width and a reduction of upper face width. When the effect of size is removed, the relationship remains equal (RV = 0.15; p<0.05) and the first pair of singular axes accounts for 37.2% of the covariance (Fig. S1). In this case, as in *Pan* and *Gorilla*, when the data are corrected for the effects of allometry, it does not substantially affect the patterns of integration. Thus, for reasons of clarity, PLS graphs without the effect of size are presented in the supplementary data.

#### *Pan*

**Figure 8:**
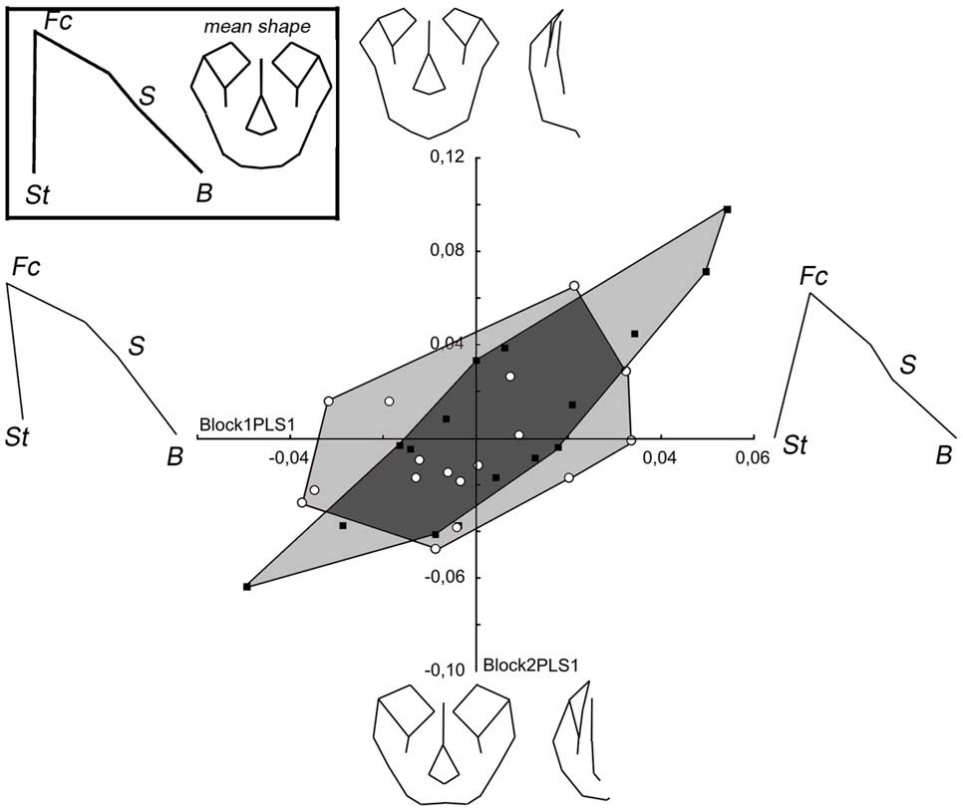
PLS of block 1 (facial block orientation) and 2 (facial shape) in *Pan*. For legend see figure 4.

The first pair of singular axes accounts for 48.3% of the total covariance in the PLS of the *Pan* specimens (Fig. 8). The relationship between the two blocks is significant and stronger than is observed for modern humans (RV = 0.31; p<0.01). For chimpanzees, increasing values indicate a ventral rotation of the facial block and a superoposterior displacement of the basion, and these are associated with a downward displacement of the lower face, an upper displacement of the middle face associated in an augmentation of the height of the piriform aperture, and also a reduction of orbit size relative to the face. Even when the size effect is removed, the correlation remains significant (RV = 0.28; p<0.05). The first pair of singular axes accounts for 45.5% of the total covariance (Fig. S2).

#### *Gorilla*

**Figure 9:**
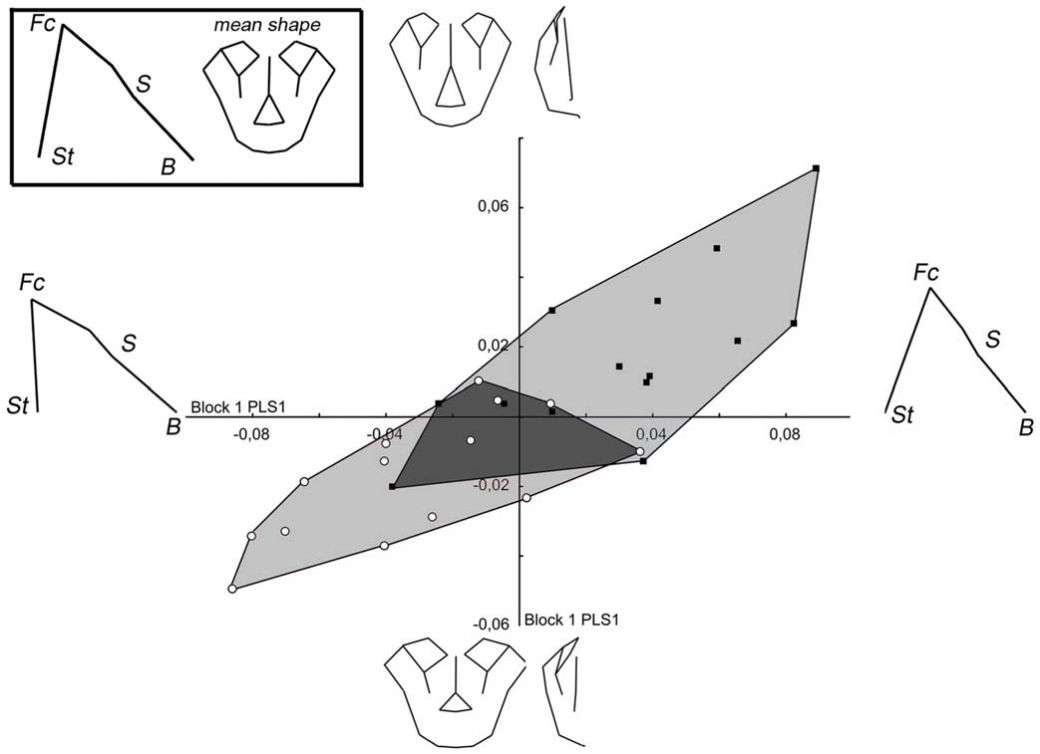
PLS of block 1 (facial block orientation) and 2 (facial shape) in *Gorilla*. For legend see figure 4.

In the PLS of *Gorilla* specimens, the first pair of singular axes accounts for 44.0% of the covariance (Fig. 9). The covariation between the two blocks is significant and stronger than for the two other taxa (RV = 0.37; p<0.01). For this species, male and female specimens are clearly separated. Concerning morphological relationships, positive scores indicate an increasing ventral rotation and an increase of the height of the facial block and an upward displacement of the basion, which is associated with a downward displacement of the lower points of the piriform aperture (nasospinale and alare) relative to the lower face, a reduction of the upper face width and an upward displacement of the orbits, infraorbital foramena, nasion and rhinion. When the effect of is size are removed, the relationship remains significant (RV = 0.35; p<0.01) and the first pair of singular axes accounts for 43.8% of the covariance (Fig. S3).

## Discussion

### Comparison of PM plane and midsagittal landmarks

In order to study the orientation of the facial block in the midsagittal plane, we choose to use midsagittal landmarks rather than the PM plane which is a midsagittal projection of lateral landmarks [8], [20]. Our results show that the angular relationship between the PM plane and the neutral horizontal axis of the orbits is significantly different from the relationship between the staphylion-sphenoidale segment and the axis of the orbits, at least for *Homo* and *Pan*. For *Gorilla*, the relationship is at the limit of statistical significance. This difference between *Homo* and *Pan* on one hand and *Gorilla* on the other hand may be explain by the peculiar palatal structure of *Gorilla*, described as very derived [66]. This confirmed that lateral parts of the basicranium, i.e, PM plane, and midsagittal parts of the basicranium, i.e, staphylion-sphenoidale, interact in different ways with the face, i.e., the axis of the orbits [13], [14] and thus, that the study of the correlation between the facial shape and the orientation of the facial block in the midsagittal plane is of prime interest. Our measurements are in accordance with the assessment that the value of the angle between the PM plane and the axis of the orbits is not significantly different across the three taxa [8], [25]. Considering our midsagittal points, the value of the angle between the staphylion-foramen caecum segment and the axis of the orbits is also not significantly different across the three taxa. At the contrary, the angle between the staphylion-sphenoidale segment and the axis of the orbits is significantly different. This shows that morphological modifications of the anterior cranial base (length, orientation or shape) during the respective evolution of the three studied taxa affect the way the posterior part of the facial block is oriented.

### Relationship between facial block orientation and cranial base flexion

The results of the first PCA including modern human specimens are consistent with Lieberman and colleagues' [3] hypothesis of an association between the reduction of the CBA value and downward rotation of the facial block. We have found that relationship exists also, to a lesser extant, in *Pan*. Nevertheless, there is no significant change in the cranial base flexion in *Gorilla* and thus, no significant relationship with the facial block. In modern humans, the augmentation of the basicranial flexion [9], [10], [67] is the product of two relative displacements, i.e. a ventral rotation of the PCF and a ventral rotation of the ACF. These displacements also influence the overall basicranium orientation relative to the facial block [68], [69]. It has been noted that, as the CBA is a classic and straightforward measurement, little attention has been paid to the difference between basicranial flexion and basicranial orientation relative to the facial block in previous studies, notably those using angles and linear measurements [70], [71]. In this study, the use of GM permits us to observe that the landmarks which represent basicranial structures vary, not only in their relative angulations, but also by antero-posterior and supero-inferior shifts or translations [12], [22], [70]. Using midsagittal landmarks instead of the projection of lateral landmarks (PM plane) [8], [20], our study is in accordance with Lieberman and colleagues' [3] hypothesis.

The comparison of the wireframes from PCA_Hom, PCA_Pan and PCA_Gor highlights the fact that *Homo* is the only genus with a staphylion (posterior palate) that is consistently situated behind the foramen caecum, and thus, a posterior face which lies almost completely beneath the ACF [67], [72]. This feature, specific to *Homo*, has been used to hypothesize that the strong relationship between cranial base and face may be specific to humans [3]. However, our results on PCA_Pan show that this relationship exists also, to a lesser extant, in chimpanzees. In this taxon, as in modern humans, the CBA reduction is due either to ventral rotation of the posterior cranial base (PC1), or to a ventral rotation of the anterior cranial base (PC2). On PCA_Gor, there is no significant change of the CBA on the first PC that represents a significant part of the variation (37.9%). On this axis, there is a forward displacement of the sella that, associated with the dorsal rotation of the facial block, brings the anterior and middle cranial base closer to the face. Bienvenu and colleagues [73] describe the morphology of the *Gorilla* brain as peculiar, with a long and narrow shape when compared to other great apes. This type of brain shape may explain the tendency to a more anteriorly-projected ACF and MCF not necessarily associated with a reduction of the CBA, which observed in our analyses. For the relationship between facial block orientation and basicranium PC2 of PCA_Gor shows the same as PC2 of PCA_Pan, specifically a ventral rotation of the anterior cranial base linked to a dorsal rotation of the facial block in both species.

### Role of allometry

For this study, we observe that within each taxon, shape (i.e. variation in Procrustes coordinates) is related to size, and this finding has been noted in other studies using differing sets of landmarks [30], [33], [44], [74]. In our covariation study, the RV values and the percentage of covariance explained by the first pair of singular axes remain constant - with and without the effect of allometry. However, for similar PLS scores, the changes along the PLS axes, e.g. anterior face height or basicranial flexion, remain fairly similar but are reduced after removing allometry. As an integrating factor [48], allometry seems to play a part in the strength of the covariation, i.e. level of integration, rather than on the way structures are morphologically integrated, i.e. pattern of integration [14]. It has already been noted that size plays a role in the relationships between the structures constituting the face. Thus, in great apes, orbit size is linked to facial size [75], which is correlated to body size [76]–[78]. However, even if within each taxon, a significant part of the variance is explained by allometry, the influence of variance fluctuations on the level of integration is minimal.

Among the taxa examined here, the *Gorilla* face exhibits the most variance that can be explained by allometry. It is the genus with the most differences in the pattern of integration before and after removing allometry. Allometry explains also most of the differences between males and females in *Gorilla*. This is also true to a lesser extent in chimpanzees but it is not the case in *Homo* where dimorphism is significantly explains by shape differences alone. The cranium of *Gorilla* displays a larger size variation between sexes than is observed in *Pan* or *Homo* [79]. Shea [74] proposed that similar heterochronic pattern, such as hypermophosis, leads to the differences between sexes in *Pan* and *Gorilla*. The extent of this pattern (i.e. hypermorphosis) should be related to the average size difference between males and females [51], and therefore it may be reduced in chimpanzees relative to gorillas. This could explain the greater significant percentage of *Gorilla*'s facial shape explained by size, which we have documented in our study.

### Covariation between facial shape, facial block orientation and cranial base flexion

Our results confirm that there is a significant intraspecific relationship between facial shape, facial block orientation, and basicranial flexion in hominids. We note that, with and without taking the effect of size into account, and contrary to expectations, the covariation is less significant in modern humans even if this taxon possesses a face that, during ontogeny, grows away from the cranial base only after a relatively long period of postnatal development, and which still lies close to the basicranium in adults [3], [75]. The relatively greater number of *Homo* specimens in the sample compared to *Pan* and *Gorilla* could also explain partly these differences of RV values. We also have to take into account that the variance estimated in set 1 will not necessarily match the variance estimated in set 2. Overall changes are however comparable between each species.

For the three studied taxa, the facial block ventral (upward) rotation is related to a global vertical elongation of the anterior face (facial shape). This feature is amplified in *Pan* and *Gorilla* where superoinferior elongation of the face is linked to the presence of a greatly protruding staphylion relative to the foramen caecum. This result underlines the more prognathic nature of these two taxa. For modern humans, the relationship exists but is less marked. Facial block orientation remains nearly vertical, i.e. orthognatic, even in specimens with high singular values that possess significant facial heights.

Some differences can be noted in the type of facial elongation, notably between *Pan* and *Gorilla* as chimpanzee elongation is situated in the lower face, while gorilla elongation is located more towards the middle face. This difference could be related to the particular facial pattern of *Gorilla*, i.e. an anteroposteriorly and superoinferiorly developed middle face [49], [80]. This feature plays also a part in sexual dimorphism as *Gorilla* males possess a more anterior middle face [51]. This character is exclusive to this taxon may explain the small differences in the covariation pattern between males and females in the first *Gorilla* PLS analysis.

While we have found a significant relationship between facial shape, facial block orientation, and cranial base position, it does not follow the hypothesis proposed by Enlow and Hans [19] of a vertically-elongated anterior face linked to a long and weakly-flexed basicranium. Indeed, in this study, the anterior face elongation in modern humans is linked to an increase in the basicranial flexion, while the basicranial length remains fairly constant. Other studies [12], [67], [81] have also found no support for Enlow and Hans' hypothesis [19], using 2D landmarks.

The main difference between modern humans on one hand and *Pan* and *Gorilla* on the other hand concerns the facial shape and basicranial flexion relationship. In modern humans, the elongation of the anterior face is correlated with a ventral rotation of the posterior cranial base and, in *Pan* and *Gorilla*, with a superoposterior shift of the basion. This difference can be noticed in PLS analyses with and without the effect of allometry.

We can hypothesize that the specificity of an anterior face elongation linked with a ventral rotation of the facial block and of the posterior cranial base seen in modern humans is linked to the necessity of keeping enough space between the posterior palate and the posterior cranial base for pharyngeal structures. In fact, the flexion of the cranial base results in a forward displacement of the basion, and in a reduction of the space between basion and staphylion. In modern humans, this space is already reduced compared with chimpanzees and gorillas. The ventral (upward) rotation of the facial block that accompanied the CBA flexion allows for such functions such as airflow, swallowing or vocalization. Although this space is reduced in modern humans, no study has yet demonstrated that humans have reached the upper limit of flexion circumscribed by cranial base and overall cranium structure [28], even if some functional limits might exist [9]. For now, this question remains unresolved.

Interestingly, the differences observed between modern humans and *Pan* and *Gorilla* can be expressed as an hypothesis proposed by McCarthy [28]. This hypothesis states that for a given basicranial length, the more flexed the posterior cranial base, the taller it is vertically, which is what we have confirmed in our study. Taking this observation into account, McCarthy [28] proposed that hominins and modern humans, which both have relatively shorter posterior cranial bases, may have had the posterior cranial base flexed through evolution in order to match the height of the nasomaxillary complex (anterior face). Our results support this hypothesis as *Homo* is the only genus where the basicranial flexion is associated with a superoinferior elongation of the anterior face.

Our study demonstrates a significant relationship between anterior facial shape, facial block orientation and basicranial flexion. A difficulty that remains is to elucidate the putative causality effects. For Lieberman et al. [3], “there are two major reasons to believe that the cranial base exerts a greater influence on the face than vice versa”. First, cranial base usually reaches adult size before the face. Second, most of the face grows around the cranial base. The question of the influence of the basicranium on the face is still complex and genetic, ontogenetic and developmental studies on the relationship between basicranium and face are here needed to confirm and complete Lieberman and colleagues' [3] hypothesis.

## Conclusions

Our results show a clear intraspecific covariation between facial shape, facial block orientation and basicranial flexion. However, our conclusions do not support Enlow and Hans' [19] hypothesis of a vertically elongated face being linked to a long and weakly-flexed basicranium. In our analysis, the anterior vertical elongation of modern humans is linked to an increase of the angle of basicranial flexion. *Homo*, *Pan* and *Gorilla* share similar characteristics in the relationship between facial shape and facial block orientation but they differ when the covariation of facial shape and basicranial flexion is considered. Modern humans show a specific pattern of integration, which underscores the significant role of their highly flexed cranial base within their cranial morphology. Our results corroborates Lieberman's hypothesis [3] of an association between reduction of the basicranial flexion and dorsal rotation of the facial block in modern humans. This relationship also exists, to a lesser extant, in *Pan* but is absent in *Gorilla*. This may be due to the particular brain shape observed in gorillas [73]. All these results highlight the fact that, along with facial size [29], facial morphology is an essential feature that must be taken into account when investigating covariation between face and basicranium.

As we have shown in this study, a clear covariation exists between basicranial flexion and facial shape in our model of extant hominids. However, cranial modifications through time in hominins suggest that characteristics such as brain volume, basicranial and facial shapes are acquired in multiple steps, implying different integration patterns for cranial architecture. The understanding of the pace of acquisition of facial and cranial characteristics during the course of evolution is essential to improve our model of covariation in the cranium. Hence, basicranial flexion level for early hominins of the late Miocene/Pliocene should be related to specific facial shapes, which differ in some ways from modern hominins whose facial characteristics evolved later [4], [82]. These difference patterns may also be due to differences in cranial functions. For example, the appearance of a new function, such as vocalization, implies modification in the pharyngeal structures [4], and therefore implies the existence of a new pattern of cranial integration, in line with this new function.

This study is the first step in a series of investigations on facial morphological variations and its relationships with the rest of the skull. Future work will expand on the present analyses, and include the mandible of each specimen in this study to clarify the role and the importance of the size and shape of the masticatory apparatus in the facial and the basicranium position.

## Supporting Information

Figure S1 **PLS of block 1 (facial block orientation) and 2 (facial shape) after removing allometry in** ***Homo*** **.** For legend see figure 4.
(TIF)

Figure S2 **PLS of block 1 (facial block orientation) and 2 (facial shape) after removing allometry in** ***Pan*** **.** For legend see figure 4.
(TIF)

Figure S3 **PLS of block 1 (facial block orientation) and 2 (facial shape) after removing allometry in** ***Gorilla*** **.** For legend see figure 4.
(TIF)
