# Supplementary figures and images for: Facial Orientation and Facial Shape in Extant Great Apes: A Geometric Morphometric Analysis of Covariation
Source: PLoS One. 2013 Feb 18;8(2):e57026. doi: 10.1371/journal.pone.0057026 (PMC3575493; doi:10.1371/journal.pone.0057026)

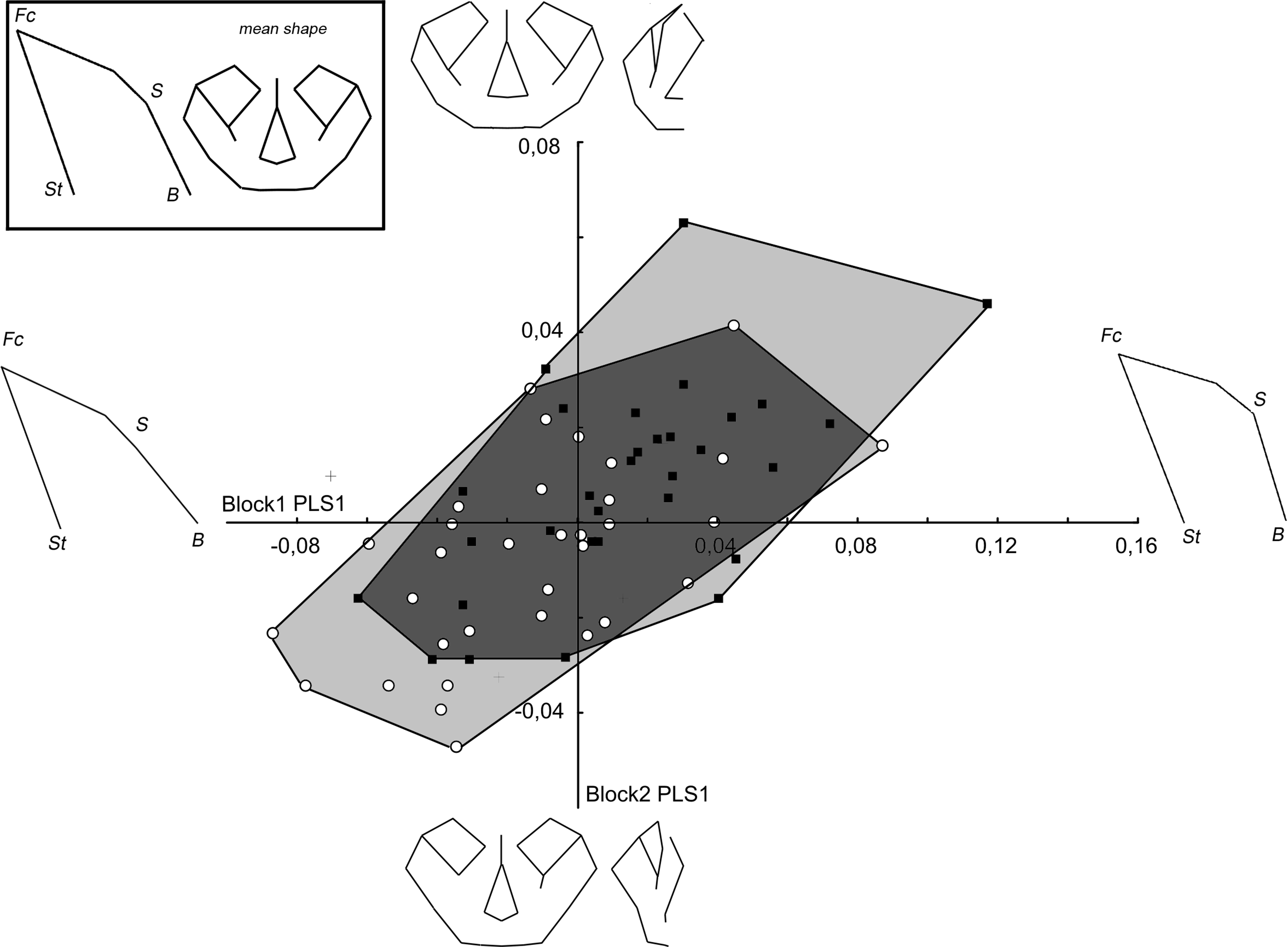

Supplement: Figure S1 — PLS of block 1 (facial block orientation) and 2 (facial shape) after removing allometry in Homo . For legend see figure 4. (TIF) [file pone.0057026.s001.tif]

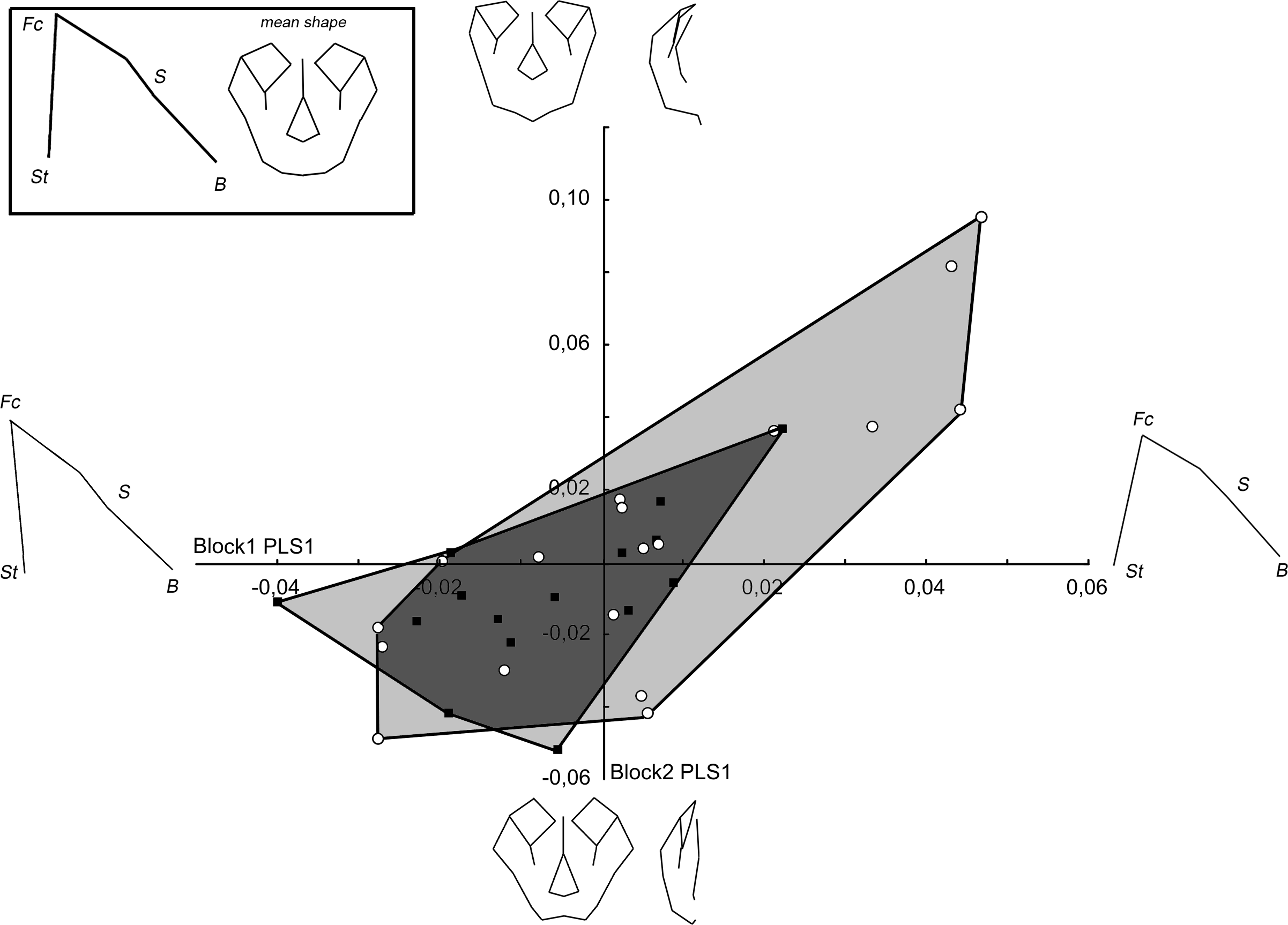

Supplement: Figure S2 — PLS of block 1 (facial block orientation) and 2 (facial shape) after removing allometry in Pan . For legend see figure 4. (TIF) [file pone.0057026.s002.tif]

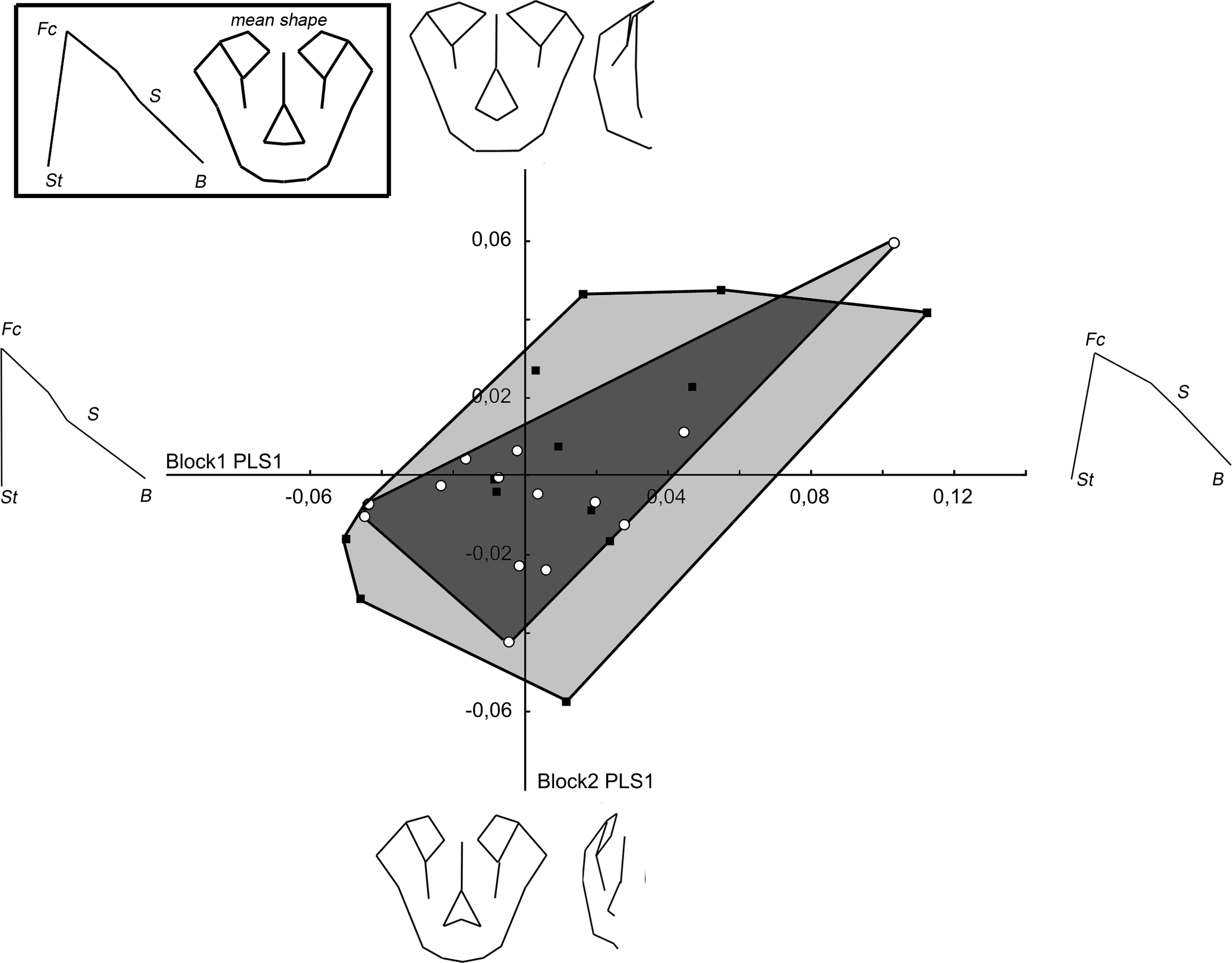

Supplement: Figure S3 — PLS of block 1 (facial block orientation) and 2 (facial shape) after removing allometry in Gorilla . For legend see figure 4. (TIF) [file pone.0057026.s003.tif]
